# Clearance of extracellular DNA prevents senescence-associated antitumor immunity

**DOI:** 10.3389/fimmu.2026.1965273

**Published:** 2026-10-14

**Authors:** Yong Wang, Lijia Yuan, Mingzhu Zhu, Mengyao Li, Qingru Lin, Yuqian Zhang, Mengwei Li, Xueyan Liu, Shun Li

**Affiliations:** 1 Department of Immunology, Shenzhen Majory Biotechnology Co., Ltd., Shenzhen, China; 2 Key Laboratory of Quantitative Synthetic Biology, Shenzhen Institute of Synthetic Biology, Shenzhen Institute of Advanced Technology, Chinese Academy of Sciences, Shenzhen, China; 3 Department of Critical Care Medicine, Shenzhen Key Laboratory of Prevention and Treatment of Severe Infections, Shenzhen People’s Hospital, First Affiliated Hospital of Southern University of Science and Technology, Second Clinical Medicine College of Jinan University, Shenzhen, China; 4 School of Life Science and Technology, China Pharmaceutical University, Nanjing, China

**Keywords:** antitumor immunity, cellular senescence, DNASE1L3, extracellular DNA, TLR9

## Abstract

**Background:**

Tumor tissues are characterized by high tumor cell turnover, driven by oncogenic proliferation and counteracted by tumor-suppressive mechanisms such as apoptosis and senescence. Dying cancer cells go through apoptosis and release apoptotic particles containing fragmented DNA—which are chromatin- or nucleosome-bound—into the tumor microenvironment. Given the immunogenicity of extracellular DNA, its efficient clearance is crucial for cancer cells to evade immune defense. However, the consequences of failed clearance of these DNA fragments on cancer cells remain poorly defined.

**Methods:**

To uncover the outcome of impaired extracellular DNA clearance in tumors, we disrupted this machinery in MMTV-PyMT spontaneous breast tumor mice by deleting both *Dnase1l3* and *Ighm* (the immunoglobulin heavy chain), thereby eliminating the DNase and its compensatory autoantibodies, and monitored tumor progression and performed histopathological, molecular, and transcriptomic profiling on harvested tumors. To determine whether cGAS or TLR9 mediates this phenotype, we intercrossed these double-deficient mice with *Sting1^-/-^* or *Tlr9^-/-^* mice and assessed antitumor immunity when extracellular DNA clearance was abrogated.

**Results:**

Combined deficiency of Dnase1l3 and anti-DNA autoantibody led to profound suppression of tumor growth in the MMTV-PyMT model. This antitumor effect was associated with senescence induction in cancer cells triggered by accumulation of uncleared extracellular chromatin DNA. Mechanistically, the senescence-associated antitumor immune response was driven by the cancer cell-intrinsic TLR9, but not cGAS.

**Conclusions:**

Our findings establish that combined deficiency of Dnase1l3 and anti-DNA autoantibodies disrupts extracellular DNA clearance and elicits a TLR9-dependent senescence-associated antitumor immune response. This pathway may represent a tissue-level immunotherapeutic strategy for cancer.

## 1 Introduction

The recognition of DNA as an immune-stimulatory molecule is an evolutionarily conserved mechanism against pathogenic infections (1). DNA usually resides in the nucleus and mitochondria in eukaryotic cells. Aberrant localization of DNA within the cytosol or endosomal compartments indicates that a cell may either be infected by a DNA virus or bacterium, or have problems with its own nuclear integrity (2). Accordingly, eukaryotic organisms have evolved several mechanisms to safeguard genomic stability (3). Clearance and sensing of inappropriately localized DNA are such defense mechanisms. For instance, DNase2 and the 3′-5′ exonuclease Trex1 (DNase3) digest mislocated cytosolic DNA to avoid excessive immune or inflammatory responses (4, 5). Even though cytosolic DNA escapes degradation by intracellular DNases and accumulates due to increased genomic instability driven by oncogenic stress, the cytosolic DNA sensor cGAS senses it and induces a senescence-associated antitumor immune response to protect against genomic insults (6). While intracellular DNA clearance and sensing mechanisms have been well characterized, extracellular DNA clearance and its associated sensing remain poorly understood. Dnase1l3 was reported to be the sole DNase containing a positively charged C-terminal tail that confers a unique property to degrade chromatin- or nucleosome-bound DNA (7). Hence, we sought to explore how undigested chromatin-bound DNA affects tumor progression in a *Dnase1l3*-null environment.

## 2 Methods

### 2.1 Mice

*Dnase1l3^-/-^* (C57BL/6JCya-*Dnase1l3^em1^*/Cya) mice, *Rag1^-/-^* (C57BL/6JCya-*Rag1^em1^*/Cya) mice, *Sting1^-/-^* (C57BL/6JCya-*Sting1^em1^*/Cya) mice, *Tlr9^-/-^* (C57BL/6JCya-*Tlr9^em1^*/Cya) mice, *Tlr9^fl/fl^* (C57BL/6JCya-*Tlr9^em1flox^*/Cya) mice, *MMTV-cre* mice, *Cd11c-cre* mice and *Cx3cr1-cre* mice were purchased from Cyagen Biosciences; *Ighm^-/-^* (C57BL/6JGpt-*Ighm^em4Cd14d43in1^*/Gpt) mice were obtained from GemPharmatech; MMTV-PyMT (PyMT) mice were obtained from Beijing Viewsolid biotech.The spontaneous mammary tumorigenic PyMT mice were crossed with *Dnase1l3^-/-^*, *Ighm^-/-^*, *Rag1^-/-^*, *Sting1^-/-^*, *Tlr9^-/-^* or *Tlr9^fl/fl^* mice to generate *Dnase1l3^-/-^*PyMT, *Ighm^-/-^*PyMT, *Rag1^-/-^*PyMT, *Sting1^-/-^*PyMT, *Tlr9^-/-^*PyMT and *Tlr9^fl/fl^*PyMT mice, respectively. *Dnase1l3^-/-^*PyMT mice and *Ighm^-/-^* or *Rag1^-/-^* mice were bred to obtain *Dnase1l3^-/-^Ighm^-/-^*PyMT and *Dnase1l3^-/-^Rag1^-/-^*PyMT mice. *Tlr9^fl/fl^*PyMT mice were crossed with *MMTV-cre*, *Cd11c-cre*, or *Cx3cr1-cre* mice to generate *MMTV-cre*;*Tlr9^fl/fl^*PyMT, *Cd11c-cre*;*Tlr9^fl/fl^*PyMT and *Cx3cr1-cre*;*Tlr9^fl/fl^* PyMT mice. *Dnase1l3^-/-^Ighm^-/-^*PyMT mice were crossed with *MMTV-cre*;*Tlr9^fl/fl^*PyMT, *Cd11c-cre*;*Tlr9^fl/fl^*PyMT and *Cx3cr1-cre*;*Tlr9^fl/fl^* PyMT mice to obtain *MMTV-cre*;*Tlr9^fl/fl^Dnase1l3^-/-^Ighm^-/-^*PyMT, *Cd11c-cre*;*Tlr9^fl/fl^Dnase1l3^-/-^Ighm^-/-^*PyMT and *Cx3cr1-cre*;*Tlr9^fl/fl^Dnase1l3^-/-^Ighm^-/-^*PyMT mice. *Dnase1l3^-/-^Ighm^-/-^*PyMT mice were crossed with *Sting1^-/-^* and *Tlr9^-/-^* mice to get *Sting1^-/-^Dnase1l3^-/-^Ighm^-/-^* and *Tlr9^-/-^Dnase1l3^-/-^Ighm^-/-^* triple knockout strain. A comprehensive summary of all mouse strains used in this study, including the abbreviations used in the figures, the targeted compartments, and the sources, is provided in **Supplementary Table 2**. All mice were backcrossed to the C57BL/6J background and maintained under specific pathogen-free conditions. Animal experiments were conducted according to guidelines approved by the Institutional Animal Care and Use Committee of Shenzhen Majory Biotechnology Co., Ltd.

### 2.2 Tumor measurement

Mammary tumors in female PyMT mice were measured weekly with a caliper. Tumor burden was calculated according to the equation [(L × W^2^) × (π/6)], where L and W denoted tumor length and width (8). Total tumor burden was calculated by summing up individual tumor volumes of each mouse, and a humane endpoint was defined when total burden reached 3000 mm^3^ or one tumor reached 2000 mm^3^.

### 2.3 Antibody cloning

DNA fragments of the 3H9 VH and VL domains were synthesized by Genewiz. Antibody protein fusion constructs were generated by overlapping PCR of DNA fragments encoding the 3H9 or control (mGO53, non-reactive) VH domain and a mouse IgG2a constant region domain. The 3H9 VH and VL sequences were obtained from a previous study (9). To abrogate IgG2a Fc effector functions, L234A/L235A/P329G mutations were introduced by site-directed mutagenesis (10) (Agilent Technologies).

### 2.4 Antibody expression and purification

Transient expression and purification of the antibody were described previously (11). FreeStyle HEK293-F cells (Cat. #K9000-01) were obtained from ThermoFisher Scientific. HEK 293F cells were transiently transfected with plasmids encoding 3H9 anti-DNA antibody, its LALA-PG mutant and isotype control antibody, and then cultured at appropriate conditions (100 rpm shaking, 8% CO_2_, 37 °C) for 6 days. Cell culture supernatants were collected and filtered by low-speed centrifugation and 0.45 μm filtration, diluted with a 10x binding buffer (0.2 M Na_3_PO_4_, pH 7.0) and passed through a protein A/G prepackaged gravity flow column (GE Healthcare). Antibodies were eluted with 0.1 M glycine-HCl (pH 2.7) into a neutralizing buffer (1 M Tris-HCl, pH 9.0), concentrated and buffer-exchanged into PBS (pH 7.4). Quantification of antibody concentrations was performed using spectrophotometry, and purity was analyzed by gel electrophoresis followed by Coomassie Blue staining. Antibody solutions were filtered through 0.22 μm filters, validated for low endotoxin levels using a Limulus amebocyte lysate (LAL) chromogenic endotoxin quantification kit (Thermo Scientific) prior to *in vivo* experiments.

### 2.5 3H9 anti-DNA antibody treatment

*Dnase1l3^-/-^Ighm^-/-^*PyMT mice bearing 5 × 5 mm mammary tumors were injected i.v. with 0.1 mL of 1 mg/mL 3H9 anti-DNA antibody (3H9 IgG2a), 3H9 anti-DNA antibody with L234A/L235A/P329G mutations (3H9 IgG2a-LALAPG), control antibody (Con IgG2a) or an equal volume PBS solution once a week for 6 weeks.

### 2.6 Cell depletion

For depletion of B cells, *Dnase1l3^-/-^*PyMT mice and PyMT mice bearing 5 × 5 mm mammary tumors were injected i.v. with 0.25 mg anti-mouse CD20 antibody (αCD20 IgG, clone 5D2, Genentech, Inc.) once every two weeks for 6 weeks, as indicated in the figure legends. mGO53 IgG2a was used as control IgG (Con IgG). For depletion of macrophages with clodronate, *Dnase1l3^-/-^*PyMT mice bearing 5 × 5 mm mammary tumors were injected i.v. with 0.2 mL of 5 mg/mL clodronate liposomes or control liposomes (obtained from ClodronateLiposomes.com) once weekly for 6 weeks.

### 2.7 SA-β-gal staining

Tumor tissues were frozen in O.C.T. medium (Sakura Finetek) and cut into 10 μm sections. The sections were fixed and stained with an SA-β-gal (Senescence-associated β-galactosidase) staining Kit (Cell Signaling Technology) according to the instructions (12). The slides were scanned by Panoramic Digital Slide Scanners (3DHISTECH LTD). CaseViewer, Fiji, and Illustrator software were used to analyze and further process the images.

### 2.8 EdU incorporation staining

PyMT, *Dnase1l3^-/-^*PyMT, *Ighm^-/-^*PyMT and *Dnase1l3^-/-^Ighm^-/-^*PyMT mice bearing 5 × 5 mm mammary tumors were intraperitoneally injected with 25 mg/kg EdU (Invitrogen) 4 hours before harvesting the tumor tissues. The tumor tissues were embedded in O.C.T. medium and sectioned at 10 μm. The detection of EdU incorporation was carried out using a Click-iT EdU Imaging Kit (Invitrogen) based on the manufacturer’s protocol.

### 2.9 Cancer cell isolation from tissues

Tumor tissues were minced with a razor blade, and then digested in 280 U collagenase type III (Worthington) and 4 μg/mL DNase1 (Sigma) in HBSS at 37 °C with shaking at 120 rpm for 30 minutes. The digested tissue was filtered with 70 µm strainers and centrifuged at 600 × g for 10 minutes. The pellet was resuspended in 44% Percoll (Sigma) and centrifuged at 3000 rpm for 30 minutes at 4 °C. The pellet was resuspended, transferred to a new conical tube after removal of the top layer, washed with PBS, and centrifuged at 600 × g for 15 minutes at room temperature. Washing was repeated twice, and the single-cell preparation from tumor tissue was ready. For enrichment of CD45^-^EpCAM^+^ cancer cells, CD45^+^ cells were depleted via magnetic separation using a MojoSort Mouse CD45 MicroBeads Selection Kit (BioLegend), and EpCAM^+^ cells were selected via magnetic separation using MojoSort Mouse CD326 (EpCAM) Selection Kit (BioLegend) following the manufacturer’s instructions.

### 2.10 Cancer cells *ex vivo* proliferation assay

CD45^-^EpCAM^+^ cancer cells isolated from PyMT, *Sting1^-/-^*PyMT and *Tlr9^-/-^*PyMT mice, were plated into 96-well plates at 2 × 10^3^ cells/well, then were grown for at least 96 hours. Cell counting kit-8 (CCK-8) (#CK04, Dojindo Laboratories) solution was added into the wells, followed by a 2-hour incubation before measurement of the absorbance at 450 nm.

### 2.11 Western blot

The isolated cancer cells (CD45^-^EpCAM^+^) from PyMT, *Dnase1l3^-/-^*PyMT, *Ighm^-/-^*PyMT and *Dnase1l3^-/-^Ighm^-/-^*PyMT mice bearing 8 × 8 mm mammary tumors were lysed in ice-cold RIPA buffer containing protease and phosphatase inhibitors (1% PMSF, 1% EDTA, 1% protease inhibitor cocktail). After protein quantification, equal amounts of protein were separated on 10% SDS-PAGE gels and blotted onto 0.45 μm PVDF membrane (Millipore). After blocking by 5% non-fat milk in TBST for 1.5 hours, membranes were incubated with the indicated primary antibodies at 4 °C overnight. Membranes were washed twice with TBST and incubated with horseradish peroxidase (HRP)-conjugated secondary antibodies at room temperature for 1 hour. Immunoblots were detected using an imaging system (Bio-Rad, USA). Primary antibodies were incubated with the membranes at the indicated dilution: β-actin (Cell Signaling Technology; 1:2000), p15 (Invitrogen, 1:1000), p16 (Abcam, 1:1000), p21 (Cell Signaling Technology, 1:1000), p27 (Cell Signaling Technology, 1:1000), CDK2 (Cell Signaling Technology, 1:1000), CDK4 (Invitrogen, 1:1000), CyclinD1 (Cell Signaling Technology, 1:1000), Cyclin E (Invitrogen, 1:1000), ERK1/2 (Cell Signaling Technology, 1:1000), Phospho-ERK1/2 (Cell Signaling Technology, 1:1000), p38 (Cell Signaling Technology, 1:1000), Phospho-p38 (Cell Signaling Technology, 1:1000), p65 (Cell Signaling Technology, 1:1000) and Phospho-p65 (Cell Signaling Technology, 1:1000).

### 2.12 Flow cytometry

Fluorescent-dye conjugated antibodies against mouse CD16/32 (2.4G2), CD45 (30-F11), B220 (RA3-6B2) and CD138 (281-2) were purchased from BioLegend. The single-cell suspensions were stained with anti-CD16/32 antibody (Fc-block) and antibodies against cell surface markers on ice for 30 minutes. All data were acquired using a CytoFLEX-S flow cytometer (Beckman Coulter) and analyzed by FlowJo software (Tree Star).

### 2.13 Chromatin (Poly-nucleosome) DNA isolation from HeLa cells

The method for poly-nucleosome isolation from HeLa cells was described previously (13). 5 × 10^8^ HeLa cells (>95% viability) in culture medium were collected by centrifugation and gently washed in PBS supplemented with 1 g MgCl_2_/L twice, and then collected as before. The cell pellet was resuspended in 15 mL of Buffer A (10 mM MES pH 6.5, 5 mM MgCl_2_, 1 mM CaCl_2_, 15 mM NaCl, 60 mM KCl, 0.25 M sucrose, 0.5% Triton X-100, 0.5 mM sodium metabisulfite, 0.5 mM benzamidine-HCl, 0.1 mM PMSF, 0.5 mM DTT, 0.5 μg/mL leupeptin, 0.7 μg/mL pepstatin, 1 μg/mL aprotinin) per 5 × 10^8^ cells and aliquoted into 3–4 Dounce homogenizers (Wheaton, 357546). Each aliquot was homogenized 10 times with a type B pestle (loose). The cell suspension was incubated on ice for 15 minutes to allow for the release of nuclei. Nuclei were collected by centrifugation at 5000 rpm for 10 minutes and washed once with Buffer A before resuspension in 1.2 mL of Buffer B (10 mM PIPES pH 6.8, 50 mM NaCl, 5 mM MgCl_2_, 1 mM CaCl_2_, 0.5 mM sodium metabisulfite, 0.5 mM benzamidine-HCl, 0.1 mM PMSF, 0.5 mM DTT, 0.5 μg/mL leupeptin, 0.7 μg/mL pepstatin, 1 μg/mL aprotinin) per 5 × 10^8^ cells. The CaCl_2_ concentration for the nuclei suspension was adjusted to 5 mM with 1 M CaCl_2_ and the suspension was pre-warmed to 37 °C for 15 minutes in a water bath. Micrococcal nuclease was added at 8 μL/mL nuclei and the suspension was digested for the optimized time (20 min at 37 °C, determined by time-course trial to achieve partial digestion and preserve poly-nucleosomes). The reaction was stopped by addition of 0.5 M EDTA to a final concentration of 4 mM. The digested nuclei were centrifuged at 4500 rpm for 5 minutes and then the pellet was resuspended in 0.5 mL nucleosome extraction buffer (20 mM Tris-HCl pH 7.9, 10 mM EDTA, 0.5 M NaCl) per 5 × 10^8^ cells and incubated on ice for 15 minutes. The digested nuclei were centrifuged at 10000 rpm for 10 minutes and the supernatant was saved. The extraction was Repeated 2–3 times, and the supernatants enriched for nucleosomes were pooled. The concentration of the extracted poly-nucleosomes was determined by adding 10 μL of pooled supernatants to 1 mL of 1M NaOH and measuring the absorbance at 260 nm using a tabletop spectrophotometer. A reading of 0.1 is designated as 1 unit. The total units are calculated as volume (mL) × A_260_ × 10.

### 2.14 ELISA

Anti-DNA and anti-nucleosome IgG were determined by ELISA using plates pre-coated with poly-L-lysine (0.05 mg/mL) for 2 hours at room temperature and then coated with 0.1 mg/mL calf thymus DNA (MedChemExpress) or 200 U/mL chromatin-bound DNA isolated from HeLa cells as antigens. After incubation with sera, the amount of bound IgG was measured with peroxidase-conjugated goat anti-mouse IgG (Proteintech) antibody. Anti-DNA or nucleosome IgG titers were determined using serial dilution of the serum from a positive animal (a Dnase1l3^-/-^ mouse) as a standard, and expressed as units per volume. Anti-DNA or anti-nucleosome IgG isotypes were measured by ELISA using calf thymus DNA as an antigen, and peroxidase-conjugated goat anti-mouse IgG, subclass IgG1, IgG2a, IgG2b and IgG3 (Proteintech). Total IgG levels in the serum were determined by ELISA using peroxidase-conjugated antibodies to IgG isotypes (Proteintech). For 3H9 anti-DNA antibody function validation, calf thymus DNA, chromatin DNA, recombinant mouse FcγRI and FcγRIII (Sino Biological) were used as antigens. Plate absorbance at 450 nm with background correction at 570 nm was detected with a MR-96A Microplate Reader (Mindray).

### 2.15 Immunofluorescence staining

Antibodies against E-cadherin (DECMA-1, 1:200), CD31 (390, 1:100), CD45 (30F-11, 1:200), IgG (Poly4053, 1:200), CD11c (N418, 1:100), VCAM-1(429(MVCAM.A), 1:100), F4/80 (BM8, 1:200), LAMP1 (1D4B, 1:100) were obtained from BioLegend. Antibodies against TLR9 (S18025A, 1:100) were purchased from Invitrogen. Antibodies against Dnase1l3 (1A4D4, 1:100) were obtained from Proteintech. Antibodies against Cleaved Caspase-3 (Cat:9661, 1:200) were purchased from Cell Signaling Technology. Tumor tissues were frozen in O.C.T. medium and sectioned at the thickness of 10 μm. The sections were fixed and stained with primary antibodies for 1 h at room temperature. Where secondary antibodies were required, sections were then incubated with fluorescently labeled secondary antibodies for 1 h at room temperature. The slides were scanned and analyzed in the same manner as described before.

### 2.16 Quantitative RT-PCR

Total RNA of the selected cancer cells (CD45^-^EpCAM^+^) from PyMT, *Dnase1l3^-/-^* PyMT, *Ighm^-/-^*PyMT, and *Dnase1l3^-/-^Ighm^-/-^*PyMT mice bearing 8 × 8 mm mammary tumors were extracted with the RNeasy Micro Kit (Qiagen), reversed-transcribed with SuperScript II Reverse Transcriptase (ThermoFisher), and amplified in a StepOnePlus Real-Time PCR System with SYBR Green PCR Master Mix (Applied Biosystems). The change-in-cycling-threshold (2^−ΔΔCt^) method was used for calculation of relative target gene expression normalized to the housekeeping transcript GAPDH. RT-PCR primer pairs included: IL-6 forward, ttccatccagttgccttcttg; reverse, aattaagcctccgacttgtgaa; TNF-α forward, aaaccaccaagtggaggagc; reverse, gcagccttgtcccttgaaga; IL-1β forward, gcttcaggcaggcagtatca; reverse, agtcacagaggatgggctct; GAPDH forward, atggtgaaggtcggtgtgaa; reverse, cgctcctggaagatggtgat.

### 2.17 Bulk RNA-seq

For transcriptome analysis, approximately 1 × 10^6^ cancer cells (CD45^-^EpCAM^+^) isolated from PyMT, *Dnase1l3^-/-^*PyMT, *Ighm^-/-^*PyMT, and *Dnase1l3^-/-^Ighm^-/-^*PyMT mice bearing 8 × 8 mm mammary tumor were collected (n = 3 biological replicates per group) and used for RNA extraction. The RNA-seq libraries were constructed by HaploX Genomics Center according to the standard protocol. High-throughput transcriptome sequencing was carried out using Illumina PE150 according to the manufacturer’s instructions. Simply, raw reads were filtered and trimmed using fastp (v0.23.0) with the base quality value > 20. The clean reads were aligned and estimated by using HISAT2 (v2.1.0) and HTSeq (v0.10.0). The levels of gene expression were quantitated as transcripts per kilobase of exon model per million mapped reads (TPM) before the normalization of read counts, and P values were adjusted by Benjamini-Hochberg (BH) method. Differentially expressed gene was defined as |Log_2_ Fold Change|≥1 and adjusted P < 0.05. Differential expression analysis was performed using edgeR package (3.20.9). The false discovery rate (FDR) was computed using the BH procedure. Gene Set Enrichment Analysis (GSEA) was performed with GSEA software version 4.3.3.

### 2.18 Statistical analysis

All experiments were repeated at least three times except measurements of individual tumors. All statistical measurements are displayed as mean ± SEM. For comparisons of two groups, an unpaired Student’s *t*-test (two-tailed) was conducted. For paired distance comparisons, paired *t*-test (two-tailed) was conducted. For comparisons of more than two groups, data were analyzed by one-way ANOVA and results were adjusted with the Bonferroni’s correction. For multiple group comparisons with more than one independent variable, two-way ANOVA was used. Data were analyzed with GraphPad Prism software. Fluorescent imaging analysis was performed in a blinded fashion.

## 3 Results

### 3.1 Dnase1l3 and B lymphocyte double deficiency suppresses tumor growth

In the MMTV-PyMT transgenic mouse model of breast cancer in which oncogenesis is triggered by the polyoma virus middle T antigen (hereafter, PyMT), Dnase1l3 is predominantly secreted by mononuclear phagocytes, including tumor-associated macrophages (TAMs) and dendritic cells (DCs) in the tumor context (**Supplementary Figures 1A, B**), consistent with previous studies (14, 15). Notably, deletion of Dnase1l3 resulted in no obvious changes in the tumor tissue, including both cell death and vasculature development (**Supplementary Figure 1C**), and failed to inhibit mammary tumor growth (**Figure 1A**). However, Dnase1l3 deficiency led to increased serum autoantibodies, including anti-dsDNA and anti-chromatin antibodies, without significantly affecting total serum antibody level (**Figure 1B**). In line with the elevated level of serum autoantibodies, Dnase1l3 deficiency also resulted in increased IgG deposition in the tumor tissue (**Figure 1C**). Previous studies have attributed pathological functions to the autoantibodies in Dnase1l3-deficient mice (16); yet, the autoantibodies may as well participate in DNA clearance (17, 18) and provide a compensatory mechanism of DNA clearance when Dnase1l3 was unavailable. To verify this, we crossed *Dnase1l3*^-/-^PyMT mice onto the B cell-deficient (*Ighm*) background to get rid of the autoantibodies. B cell deficiency on top of Dnase1l3 loss resulted in profound tumor repression, and dramatically extended the lifespan of mice (**Figures 1D, E**). These results imply that the autoantibodies might provide a compensatory mechanism of extracellular DNA clearance during Dnase1l3 deficiency, and thus mask the antitumor properties of extracellular DNA.

**Figure 1:**
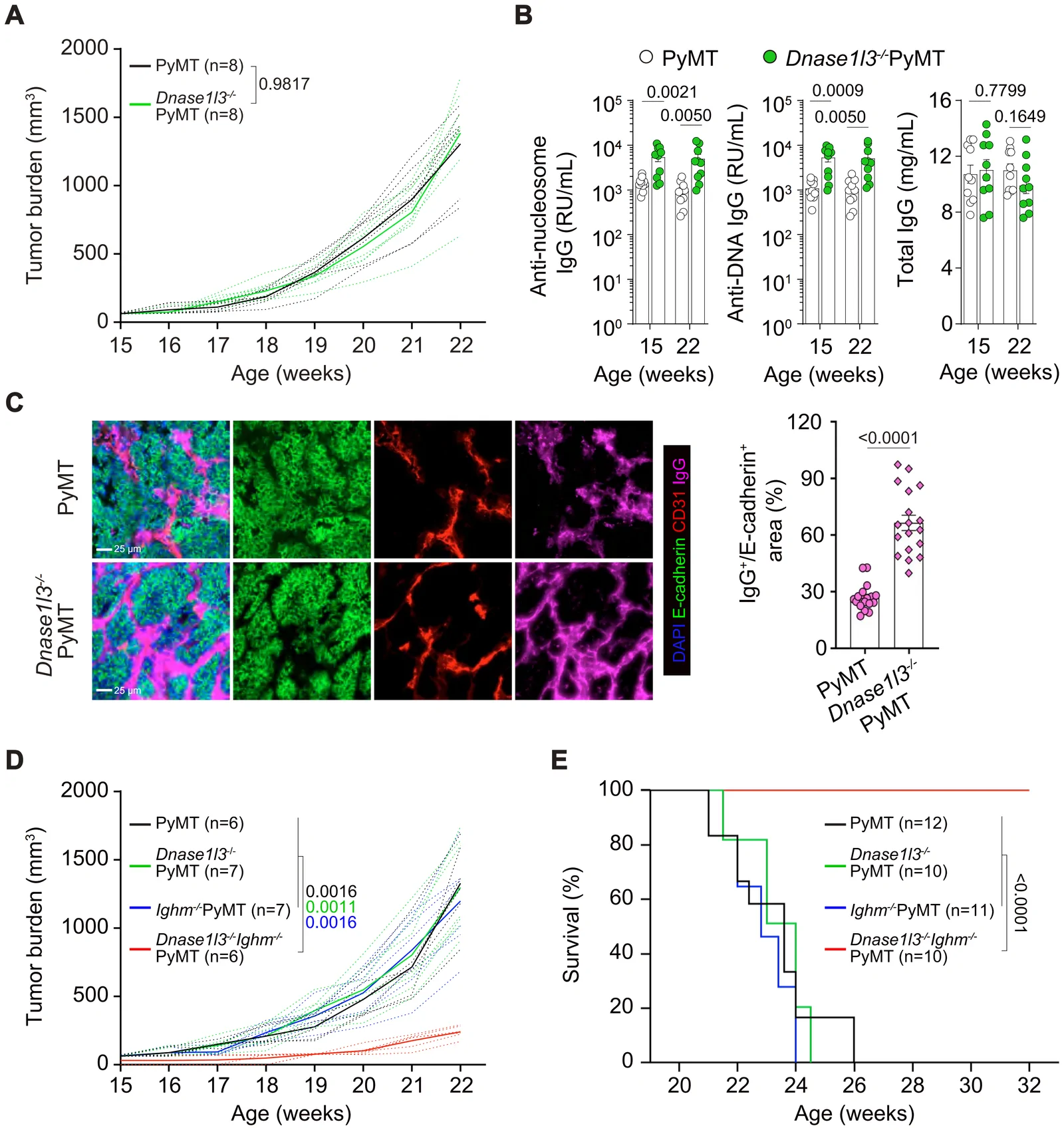
Dnase1l3 and autoantibody double deficiency retards tumor growth. **(A)** Tumor burden in PyMT and *Dnase1l3^-/-^*PyMT mice (n = 8 for each). **(B)** ELISA to assess the serum levels of anti-nucleosome IgG, anti-DNA IgG and total IgG in PyMT and *Dnase1l3^-/-^*PyMT mice (n = 10 for each group). **(C)** Representative immunofluorescence images of DAPI (blue), E-cadherin (green), CD31 (red), and IgG (magenta) in tumor tissues from PyMT and *Dnase1l3^-/-^*PyMT mice (left), and quantification of IgG deposition (right). The percentage of IgG^+^ as a proportion of total E-cadherin^+^ cells was calculated from regions of 0.04-mm^2^. **(D)** Tumor burden in PyMT, *Dnase1l3^-/-^*PyMT, *Ighm^-/-^*PyMT and *Dnase1l3^-/-^Ighm^-/-^*PyMT mice (n = 6, 7, 7 and 6, respectively). **(E)** Kaplan-Meier survival curve of PyMT, *Dnase1l3^-/-^*PyMT, *Ighm^-/-^*PyMT and *Dnase1l3^-/-^Ighm^-/-^*PyMT mice (n = 12, 10, 11 and 10, respectively). Scale bars, 25 μm. Data are mean ± SEM **(B, C)**; Two-tailed unpaired *t*-test **(A–C)**, two-way ANOVA with *post hoc* Bonferroni *t*-test **(D)** and log-rank (Mantel-Cox) test **(E)**. Data are pooled biological replicates **(A, B, D, E)** or representative of three independent experiments **(C)**.

### 3.2 Anti-DNA autoantibody compensates for Dnase1l3 loss

To clearly define the compensatory role of autoantibodies in extracellular DNA clearance, we tried to delete the autoantibodies alone while keeping other antibodies intact. Intravenous injection of αCD20 depleting antibody led to reduction of B cells in the spleen, lymph nodes, bone marrow, blood, kidney and tumor tissue in one week, while plasma cells were not affected (**Figure 2A**). Consistently, the serum level of autoantibodies was greatly reduced 2 weeks post αCD20 antibody treatment, while that of total antibody was not significantly affected (**Figures 2B**, **3A**). Notably, treatment of αCD20 antibody led to tumor growth inhibition in *Dnase1l3*^-/-^PyMT mice but not PyMT mice (**Figure 3B**). These data suggest that autoantibodies compensate for the loss of Dnase1l3 function, which prevent extracellular DNA from suppressing tumor growth. Previous studies demonstrated that *Dnase1l3*-deficient mice manifest rapid and primary autoantibodies to dsDNA and chromatin (14), which implies that the anti-DNA and anti-nucleosome autoantibodies might make up for the loss of Dnase1l3 function. In addition, anti-dsDNA and anti-chromatin antibodies in the sera of *Dnase1l3*^-/-^PyMT mice primarily comprised the more pathogenic IgG2a and IgG2b isotypes instead of the less pathogenic IgG1 or IgG3 (**Supplementary Figure 2A**). As previously reported, an anti-DNA antibody 3H9, isolated from a diseased MRL/lpr mouse, binds DNA as well as chromatin (9). To probe whether restoration of anti-DNA antibodies could reverse the tumor rejection phenotype, we engineered recombinant anti-DNA antibodies comprising complete Fc effector activities, 3H9 IgG2a. Meanwhile, the nonreactive isotype control IgG2a and the nonpathogenic mutant 3H9 IgG2a-LALAPG (null Fc function) were used as controls (**Supplementary Figures 2B, C**). Upon intravenous injection, these recombinant antibodies were readily detected in the serum with comparable half-lives (**Supplementary Figure 2D**) and diffused into the tumor vasculature and parenchyma quickly (**Figure 3C**). As shown in **Figure 3D**, 3H9 IgG2a infusion into *Dnase1l3*^-/-^*Ighm*^-/-^PyMT mice could reverse the tumor rejection phenotype, while the nonpathogenic mutant and nonreactive isotype controls failed. These data indicate that anti-DNA antibodies could compensate for the loss of Dnase1l3 function, which is dependent on its Fc effector function. In this context, the Fc-mediated effector function in DNA clearance is more likely to be related to antibody-dependent cellular phagocytosis (ADCP) (19). To verify that, we depleted macrophages to block the ADCP function. Treatment of *Dnase1l3*^-/-^PyMT mice with clodronate liposomes depleted macrophages in the tumor tissue and retarded tumor growth compared to those treated with control liposome (**Figures 3E, F**). Taken together, these results suggest that anti-DNA antibody-dependent cellular phagocytosis provides a compensatory mechanism for Dnase1l3 in extracellular DNA clearance.

**Figure 2:**
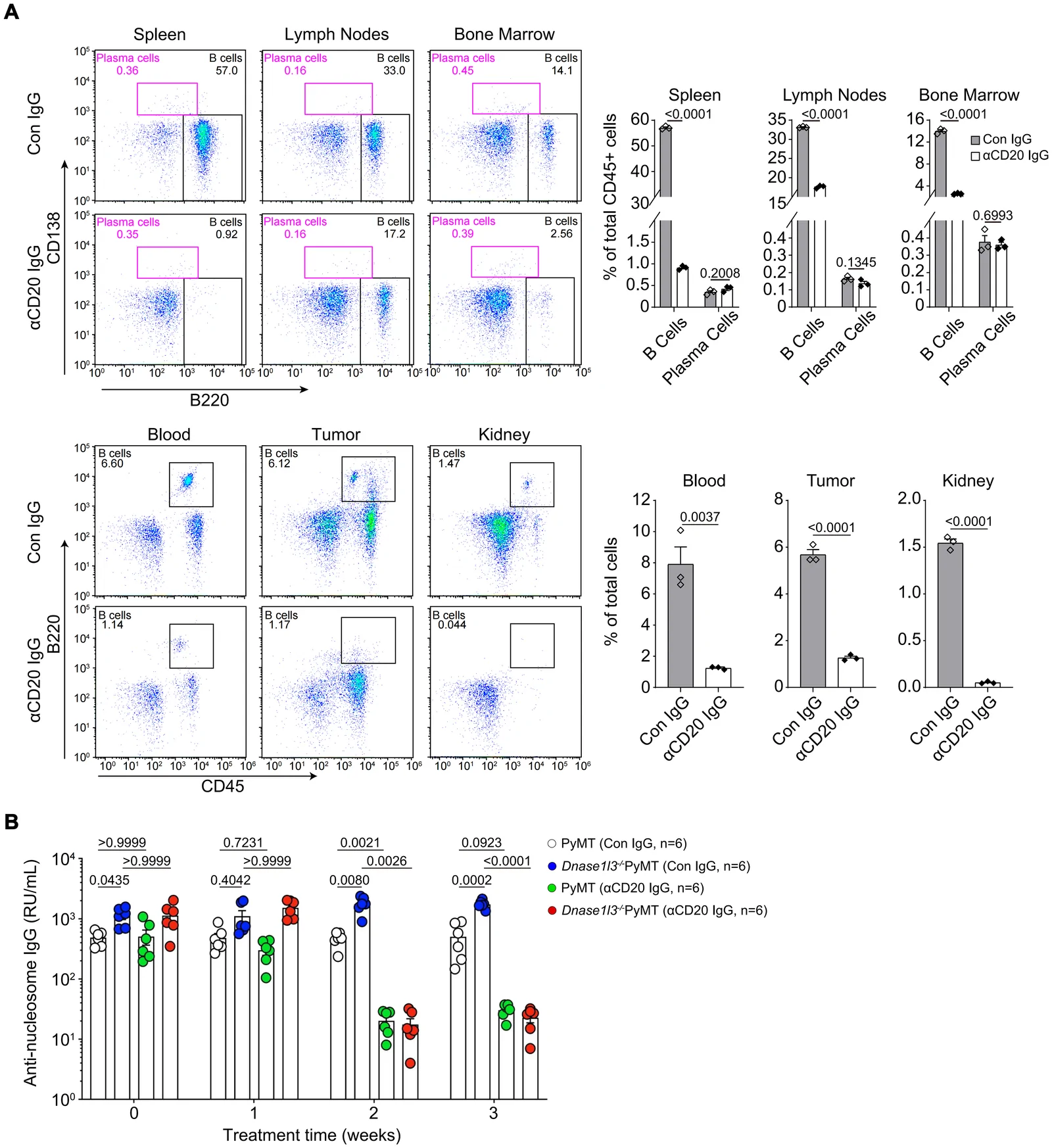
The effect of αCD20 depleting antibody treatment on B cells, plasma cells and autoantibody. **(A)** Representative flow cytometry plots and statistical analysis of CD45^+^B220^+^ B cells and CD45^+^CD138^+^ plasma cells in spleen, lymph nodes, bone marrow, blood, tumor and kidney samples from PyMT mice treated with αCD20 depleting antibody or control antibody. **(B)** ELISA to assess the serum levels of anti-nucleosome IgG in PyMT and *Dnase1l3^-/-^* PyMT mice (n = 6 for each) treated with anti-CD20 IgG (αCD20 IgG) or control IgG (Con IgG). Data are mean ± SEM **(A, B)**; Two-tailed unpaired *t*-test **(A)** and two-way ANOVA with *post hoc* Bonferroni *t*-test **(B)**. Data are pooled biological replicates **(B)** or representative of three independent experiments **(A)**.

**Figure 3:**
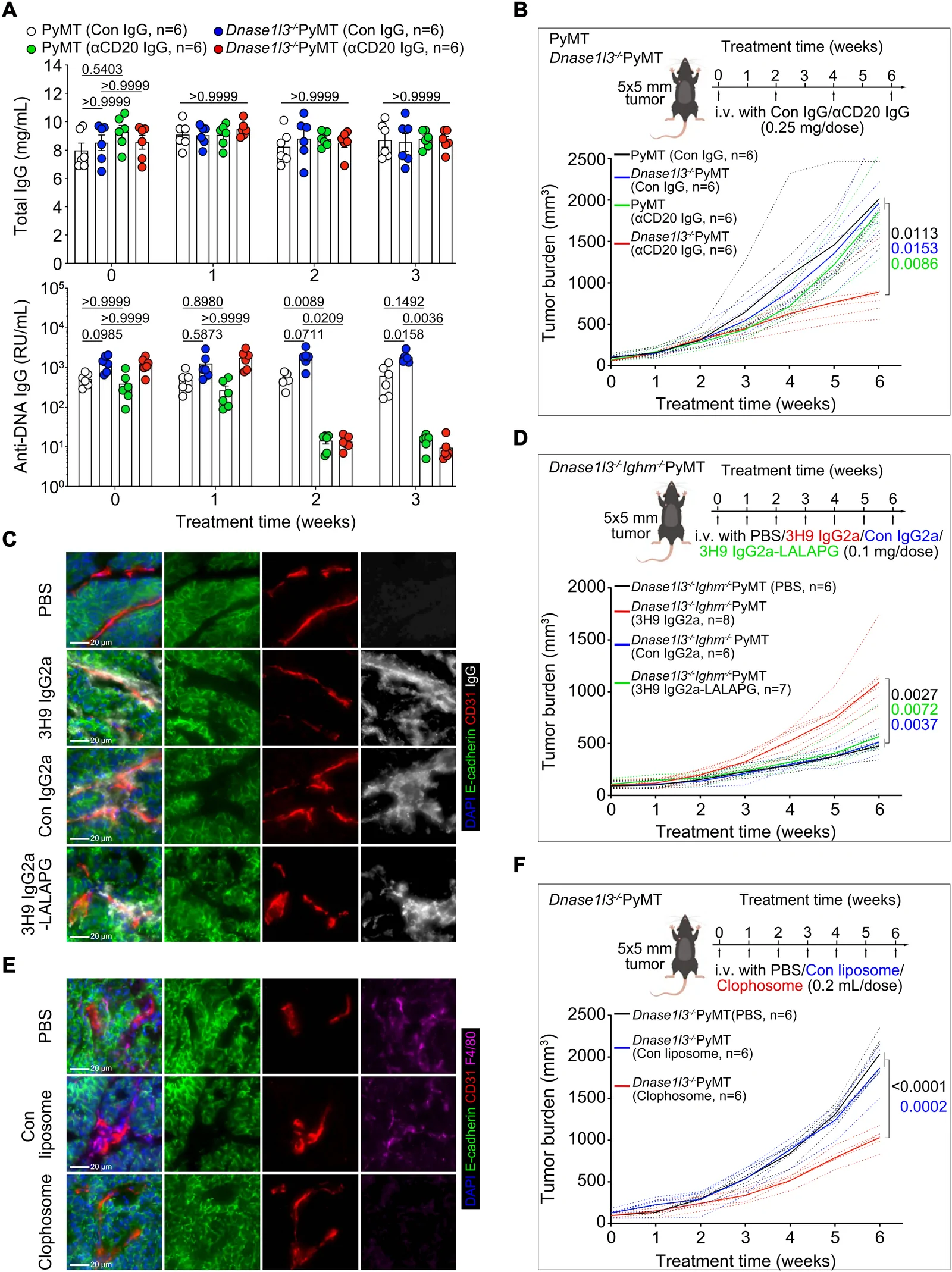
Anti-DNA antibody compensates for Dnase1l3 function loss. **(A)** ELISA to assess the serum levels of total IgG and anti-DNA IgG in PyMT and *Dnase1l3^-/-^*PyMT mice (n = 6 for each) treated with control IgG (Con IgG) or anti-CD20 IgG (αCD20 IgG). **(B)** Schematic of a treatment scheme of PyMT and *Dnase1l3^-/-^*PyMT mice bearing 5 × 5 mm tumor injected with Con IgG or αCD20 IgG (up) and tumor growth was measured (n = 6 for each group). **(C)** Representative image showing DAPI (blue), E-cadherin (green), CD31 (red) and IgG (white) immunofluorescence in tumor tissues from *Dnase1l3^-/-^Ighm^-/-^*PyMT mice treated with PBS, anti-DNA IgG2a (3H9 IgG2a), control IgG2a (Con IgG2a) or anti-DNA IgG2a with LALAPG mutation (3H9 IgG2a-LALAPG). **(D)** Schematic of a treatment scheme of *Dnase1l3^-/-^Ighm^-/-^*PyMT mice bearing 5 × 5 mm tumor treated with PBS (n = 6), 3H9 IgG2a (n = 8), Con IgG2a (n = 6) or 3H9 IgG2a-LALAPG (n = 7), respectively, and tumor growth was monitored. **(E)** Representative images showing DAPI (blue), E-cadherin (green), CD31 (red) and F4/80 (magenta) immunofluorescence in tumor tissues from *Dnase1l3^-/-^*PyMT mice treated with PBS, control liposome (Con liposome) or Clophosome. **(F)** Schematic of a treatment scheme of *Dnase1l3^-/-^*PyMT mice bearing 5 × 5 mm tumor treated with PBS, Con liposome or Clophosome (n = 6 for each group), and tumor growth was measured. Scale bars, 20 μm. Data are mean ± SEM **(A)**; Two-way ANOVA with *post hoc* Bonferroni *t*-test **(A, B, D, F)**. Data are pooled biological replicates **(A, B, D, F)** or representative of three independent experiments **(C, E)**.

### 3.3 Cancer cell senescence determines tumor suppression

To define the underlying mechanisms of tumor repression in *Dnase1l3*^-/-^*Ighm*^-/-^PyMT mice, we assessed proliferation and death of cancer cells. No difference in cell death was observed in all groups (**Supplementary Figure 3A**). Furthermore, we determined the cancer cell proliferation through pulse-labeling assays with nucleotide analog EdU incorporation (20). Notably, much less EdU incorporation was observed in the tumor from *Dnase1l3*^-/-^*Ighm*^-/-^PyMT mice compared to control groups, indicating that tumor repression in *Dnase1l3*^-/-^*Ighm*^-/-^PyMT mice was associated with reduced proliferation of cancer cells (**Figure 4A**).

**Figure 4:**
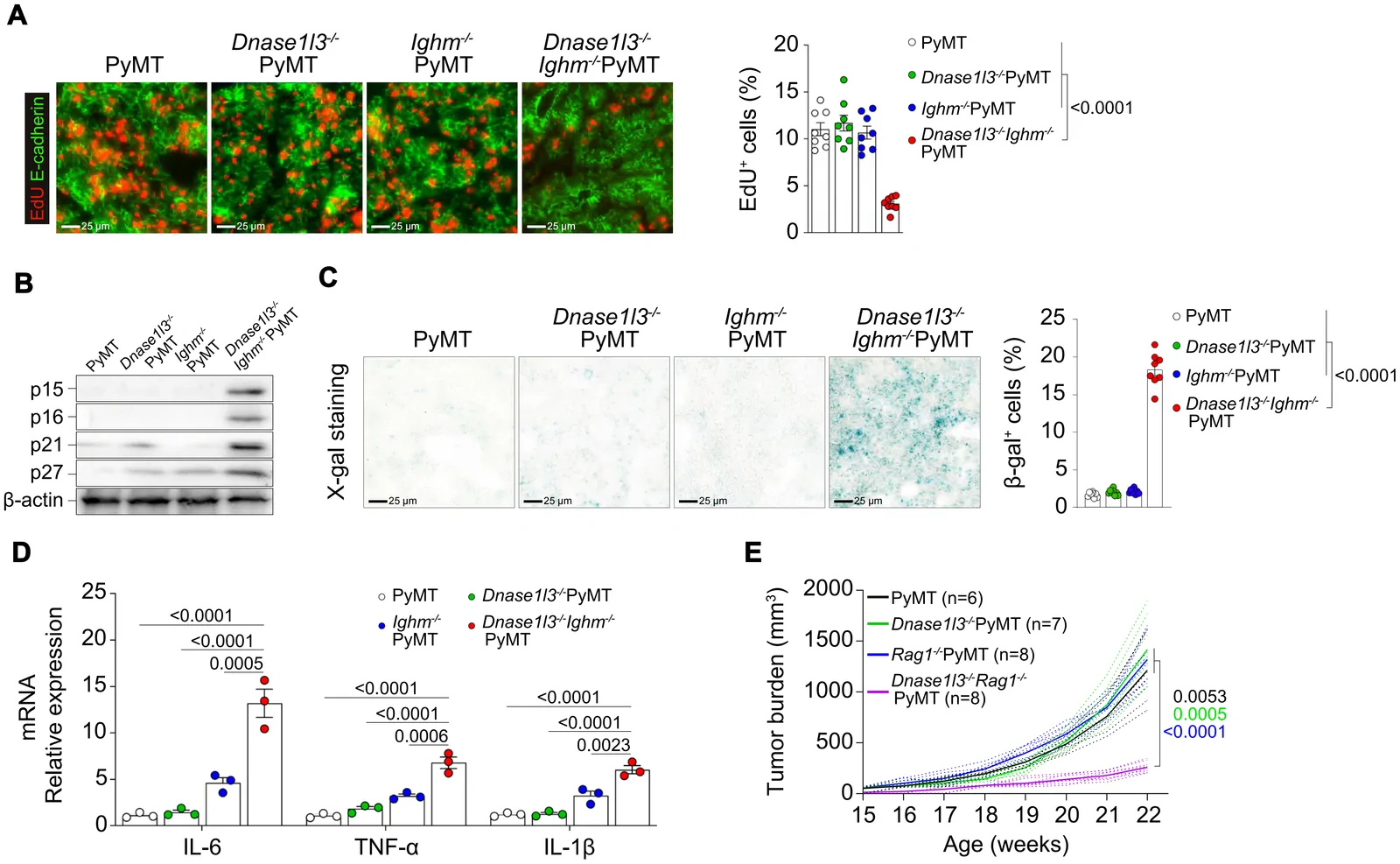
Dnase1l3 and autoantibody double deficiency induces cancer cell senescence. **(A)** Representative immunofluorescence of E-cadherin (green) and EdU (red) in tumor tissue from PyMT, *Dnase1l3^-/-^*PyMT, *Ighm^-/-^*PyMT and *Dnase1l3^-/-^Ighm^-/-^*PyMT mice. EdU^+^E-cadherin^+^ cells as a percentage of total E-cadherin^+^ epithelial cells was calculated from 0.04-mm^2^ regions (n = 8 for each group). **(B)** Immunoblot of CDKi (p15, p16, p21, p27) protein expression in the CD45^-^EpCAM^+^ cancer cells sorted from PyMT, *Dnase1l3^-/-^*PyMT, *Ighm^-/-^*PyMT and *Dnase1l3^-/-^Ighm^-/-^*PyMT mice. **(C)** Representative image of β-galactosidase (β-gal) staining in tumor tissue from PyMT, *Dnase1l3^-/-^*PyMT, *Ighm^-/-^*PyMT and *Dnase1l3^-/-^Ighm^-/-^*PyMT mice. Blue staining indicates senescent β-gal^+^ cells. The percentage of β-gal^+^ cells over total cells was calculated from 0.04-mm^2^ regions (n = 8 for each group). **(D)** Quantitative RT-PCR analysis of SASP (IL-6, TNF-α, IL-1β) mRNA expression in the CD45^-^EpCAM^+^ cancer cells sorted from PyMT, *Dnase1l3^-/-^*PyMT, *Ighm^-/-^*PyMT and *Dnase1l3^-/-^Ighm^-/-^*PyMT mice. **(E)** Tumor burden in PyMT, *Dnase1l3^-/-^*PyMT, *Rag1^-/-^*PyMT and *Dnase1l3^-/-^Rag1^-/-^*PyMT mice (n = 6, 7, 8 and 8, respectively). Scale bars, 25 μm. Data are mean ± SEM **(A, C, D)**; Two-way ANOVA with *post hoc* Bonferroni *t*-test **(A, C–E)**. Data are pooled biological replicates **(E)** or representative of three independent experiments **(A–D)**.

The reduced proliferation of cancer cells prompted us to consider whether cancer cells were arrested in the cell cycle. Successful progression through the cell cycle is mediated by a series of related proteins, termed cyclin, cyclin-dependent kinase (CDK), and CDK inhibitor (CDKi) (21, 22). No difference in the expression of Cyclins (Cyclin D1 and Cyclin E) as well as CDKs (CDK2 and CDK4) was observed among all groups (**Supplementary Figure 3B**), but CDKis (p15^Ink4B^, p16^Ink4A^, p21^Waf1^ and p27^Kip1^) were highly induced in the cancer cells from *Dnase1l3*^-/-^*Ighm*^-/-^PyMT mice (**Figure 4B**). It was reported that elevated levels of CDKi induce cellular senescence, a permanent state of cell cycle arrest (23). Notably, cancer cells in the tumor from *Dnase1l3*^-/-^*Ighm*^-/-^PyMT mice exhibited increased staining of β-gal (β-galactosidase) (**Figure 4C**) and gene transcription of senescence-associated secretory phenotype (SASP), a characteristic pro-inflammatory secretory signature of senescent cells, represented by pro-inflammatory cytokines (**Figure 4D**), two hallmarks of cellular senescence (24, 25). Consistently, in the autoantibody depletion (**Figure 3B**), restoration (**Figure 3D**), and macrophage depletion experiments (**Figure 3F**), the senescence phenotype strictly paralleled the tumor inhibition phenotype (**Supplementary Figure 4**). Taken together, these data reveal that cancer cell senescence is induced when extracellular DNA fails to be cleared by Dnase1l3 or anti-DNA antibody.

Given its immunogenic nature, the uncleared extracellular DNA will elicit the host immune responses that aim to prevent its regeneration. In this context, cancer cell senescence is such an immune response that prevents cancer cells from proliferating and producing more apoptotic DNA. To explore the essence of such senescence-associated immune response, we determined whether it is adaptive or innate immunity-dependent. Similar phenotypes, such as tumor repression, CDKi protein induction as well as cancer cell senescence, were observed when crossing *Dnase1l3*^-/-^PyMT mice to the Rag1-deficient background to delete both T cells and B cells (**Figures 4E**, **5A, B**), excluding T cells or adaptive systems as anti-tumor effectors in this senescence response. These data imply that the extracellular DNA-induced senescence response is innate immunity-dependent.

**Figure 5:**
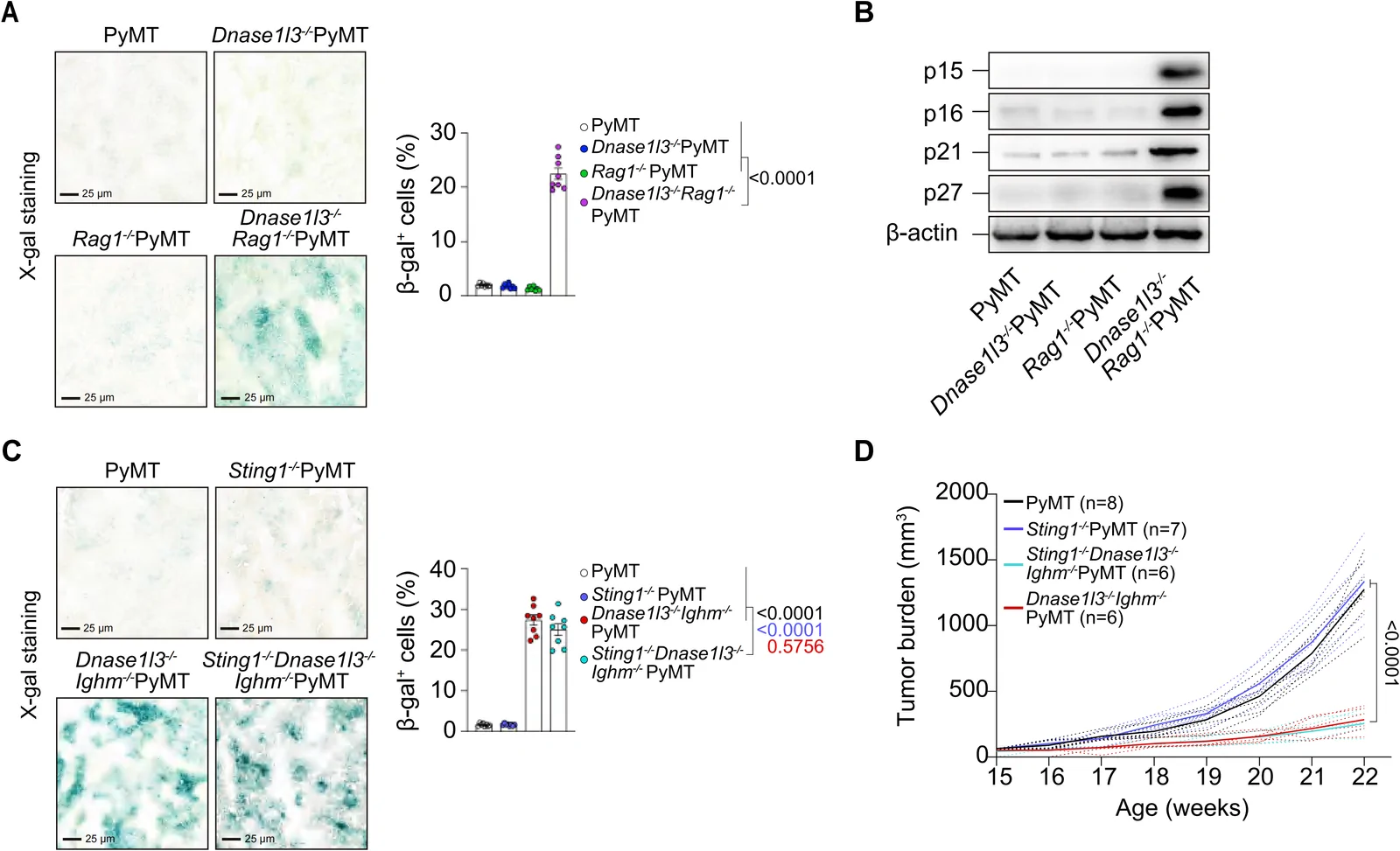
Dnase1l3 and autoantibody double deficiency mediates STING-independent cellular senescence. **(A)** Representative images of β-gal staining in tumor tissue from PyMT, *Dnase1l3^-/-^*PyMT, *Rag1^-/-^*PyMT and *Dnase1l3^-/-^Rag1^-/-^*PyMT mice. The percentage of β-gal^+^ cells over total cells was calculated from 0.04-mm^2^ regions (n = 8 for each group). **(B)** Immunoblot of CDKi (p15, p16, p21, p27) protein expression in the CD45^-^EpCAM^+^ cancer cells sorted from PyMT, *Dnase1l3^-/-^*PyMT, *Rag1^-/-^*PyMT and *Dnase1l3^-/-^Rag1^-/-^*PyMT mice. **(C)** Representative images of β-gal staining in tumor tissue from PyMT, *Sting1^-/-^*PyMT, *Dnase1l3^-/-^Ighm^-/-^*PyMT and *Sting1^-/-^Dnase1l3^-/-^Ighm^-/-^*PyMT mice. The percentage of β-gal^+^ cells over total cells was calculated from 0.04-mm^2^ regions (n = 8 for each group). **(D)** Tumor burden in PyMT, *Sting1^-/-^*PyMT, *Sting1^-/-^Dnase1l3^-/-^Ighm^-/-^*PyMT and *Dnase1l3^-/-^Ighm^-/-^*PyMT mice (n = 8, 7, 6 and 6, respectively) Scale bars, 25 μm. Data are mean ± SEM **(A, C)**; ns, not significant; Two-way ANOVA with *post hoc* Bonferroni *t*-test **(A, C, D)**. Data are pooled biological replicates **(D)** or representative of three independent experiments **(A–C)**.

### 3.4 Cancer cell-intrinsic TLR9 drives senescence

Having established that uncleared extracellular DNA induces senescence-associated innate immunity, we further probed what kind of DNA sensing pathway is involved in this innate immune response. It has been shown that uncleared self-DNA gaining access to the cytosol is recognized by the cytosolic DNA sensor cGAS (26, 27), which activates inflammatory responses and induces cellular senescence (6). Hence, we investigated whether cGAS-STING signaling is involved in sensing of extracellular DNA and triggering cancer cell senescence. Indeed, crossing *Dnase1l3*^-/-^*Ighm*^-/-^PyMT mice to a STING-deficient background failed to reverse tumor rejection as well as cancer cell senescence phenotype (**Figures 5C, D**), ruling out the role of cGAS-STING axis in the senescence-associated innate immunity. In addition, exogenous DNA is also reported to be sensed by the endolysosomal DNA sensor Toll-like receptor 9 (TLR9) (28). Notably, a much higher level of TLR9 was detected in the endolysosomes of cancer cells from *Dnase1l3*^-/-^*Ighm*^-/-^PyMT mice, compared with other counterparts (**Figures 6A, B**). To this end, we probed whether TLR9 is involved in the sensing of extracellular DNA. Indeed, crossing *Dnase1l3*^-/-^*Ighm*^-/-^PyMT mice to a TLR9-deficient background reversed the cancer cell senescence phenotype and caused tumor outgrowth (**Figures 6C, D**), suggesting that TLR9 is involved in the senescence-associated innate immunity. As TLR9 is typically expressed on immune cells, but not on epithelial-lineage cells (29) (**Figure 6E**), we next explored which source of TLR9 triggers the cancer cell senescence phenotype. Actually, specific deletion of TLR9 on cancer cells, but not macrophages or dendritic cells, phenocopied global TLR9 knockout in tumor outgrowth and senescence inhibition (**Figures 6F–H**, **I**). These data together suggest that cancer cell-intrinsic TLR9 signaling is engaged in the senescence-associated innate immunity.

**Figure 6:**
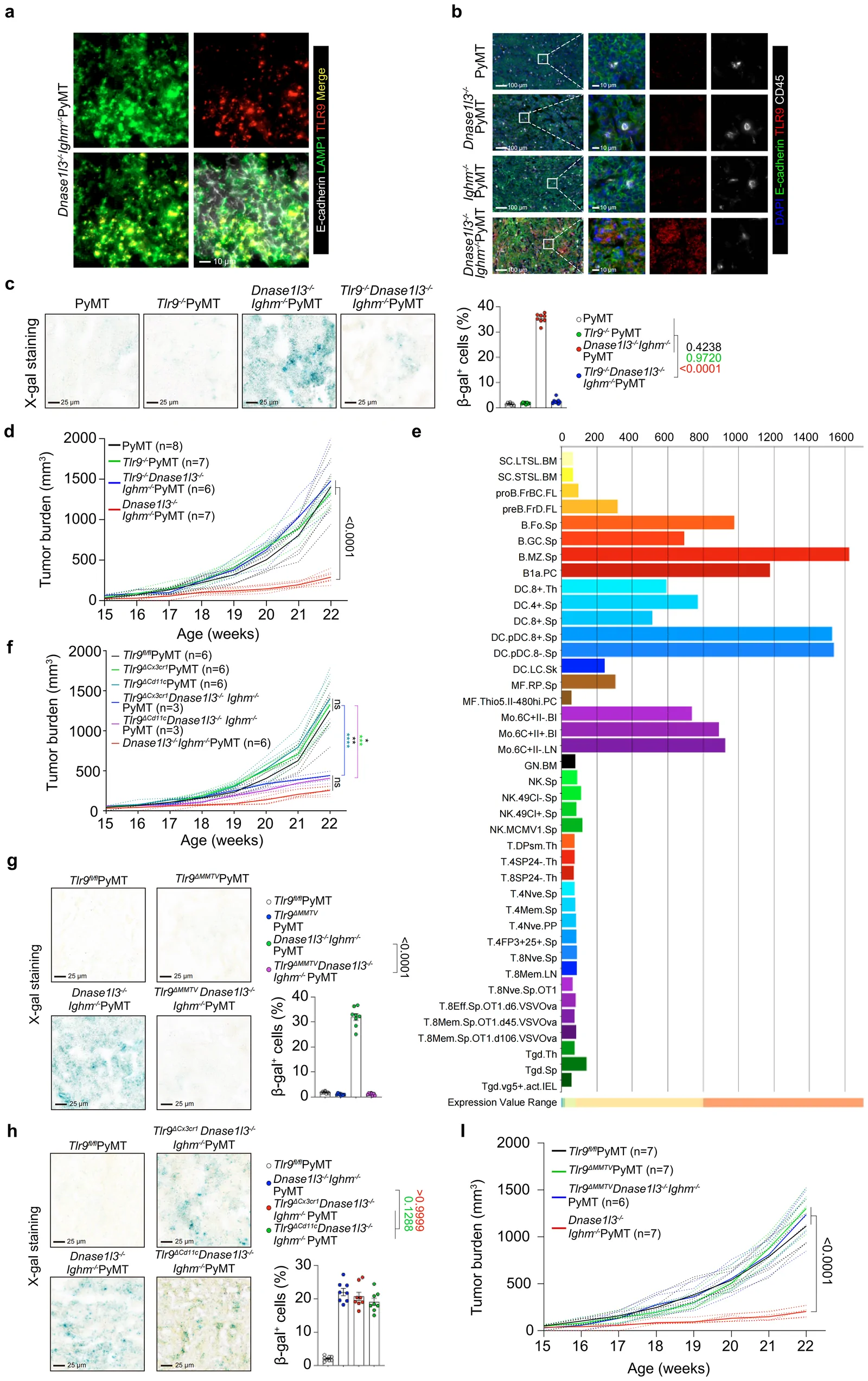
Cancer cell-intrinsic TLR9 signaling inhibits cancer cell proliferation. **(A)** Representative immunofluorescence image of E-cadherin (white), LAMP1 (green), TLR9 (red) and the merged signal of LAMP1 and TLR9 (yellow) in tumor tissue from *Dnase1l3^-/-^Ighm^-/-^*PyMT mice. **(B)** Representative immunofluorescence of DAPI (blue), E-cadherin (green), TLR9 (red) and CD45 (white) in tumor tissue from PyMT, *Dnase1l3^-/-^*PyMT, *Ighm^-/-^*PyMT and *Dnase1l3^-/-^Ighm^-/-^*PyMT mice. **(C)** Representative images of β-gal staining in tumor tissue from PyMT, *Tlr9^-/-^*PyMT, *Dnase1l3^-/-^Ighm^-/-^*PyMT and *Tlr9^-/-^Dnase1l3^-/-^Ighm^-/-^*PyMT mice. The percentage of β-gal^+^ cells over total cells was calculated from 0.04-mm^2^ regions (n = 8 for each group). **(D)** Tumor burden in PyMT, *Tlr9^-/-^*PyMT, *Tlr9^-/-^Dnase1l3^-/-^Ighm^-/-^*PyMT and *Dnase1l3^-/-^Ighm^-/-^*PyMT mice (n = 8, 7, 6 and 7, respectively). **(E)** The expression of *Tlr9* gene in murine immune cell types. Shown is the expression profile of *Tlr9* gene in the ImmGen microarray expression database of key populations with the relevant cell types indicated. **(F)** Tumor burden in *Tlr9^fl/fl^*PyMT, *Tlr9^ΔCx3cr1^*PyMT, *Tlr9^ΔCd11c^*PyMT, *Tlr9^ΔCx3cr1^Dnase1l3^-/-^Ighm^-/-^*PyMT, *Tlr9^ΔCd11c^Dnase1l3^-/-^Ighm^-/-^*PyMT and *Dnase1l3^-/-^Ighm^-/-^*PyMT mice (n = 6, 6, 6, 3, 3 and 6, respectively). **(G, H)** Representative images of β-gal staining in tumor tissue from *Tlr9^fl/fl^*PyMT, *Tlr9^ΔMMTV^*PyMT, *Tlr9^ΔMMTV^Dnase1l3^-/-^Ighm^-/-^*PyMT, *Tlr9^ΔCx3cr1^Dnase1l3^-/-^Ighm^-/-^*PyMT, *Tlr9^ΔCd11c^Dnase1l3^-/-^Ighm^-/-^*PyMT and *Dnase1l3^-/-^Ighm^-/-^*PyMT mice. **(I)** Tumor burden in *Tlr9^fl/fl^*PyMT, *Tlr9^ΔMMTV^*PyMT, *Tlr9^ΔMMTV^Dnase1l3^-/-^Ighm^-/-^*PyMT and *Dnase1l3^-/-^Ighm^-/-^*PyMT mice (n = 7, 7, 6 and 7, respectively). Scale bars, 100 μm, 25 μm and 10 μm. Data are mean ± SEM **(C, G, H)**; *P < 0.05; **P < 0.01; ***P < 0.001; ****P < 0.0001; ns: not significant; Two-way ANOVA with *post hoc* Bonferroni *t*-test **(C, D, F–I)**. Data are pooled biological replicates **(D, F, I)** or representative of three independent experiments **(A–C, G, H)**.

Furthermore, we probed whether cancer cell-intrinsic TLR9 directly senses extracellular chromatin DNA. Cancer cells isolated from PyMT mice, *Tlr9*^-/-^PyMT mice and *Sting1*^-/-^PyMT mice, displayed comparable *in vitro* cell proliferation rate (**Figure 7A**); yet, tumor cell culture with chromatin DNA, mimicking *in vivo Dnase1l3* and *Ighm* deficiency, caused cell proliferation arrest, transcriptional activation of SASP genes and further cellular senescence in cancer cells from PyMT mice and *Sting1*^-/-^PyMT mice but not *Tlr9*^-/-^PyMT mice (**Figures 7B–D**). These data reinforce that extracellular chromatin DNA could directly be sensed by TLR9 in cancer cells and cause cancer cell senescence.

**Figure 7:**
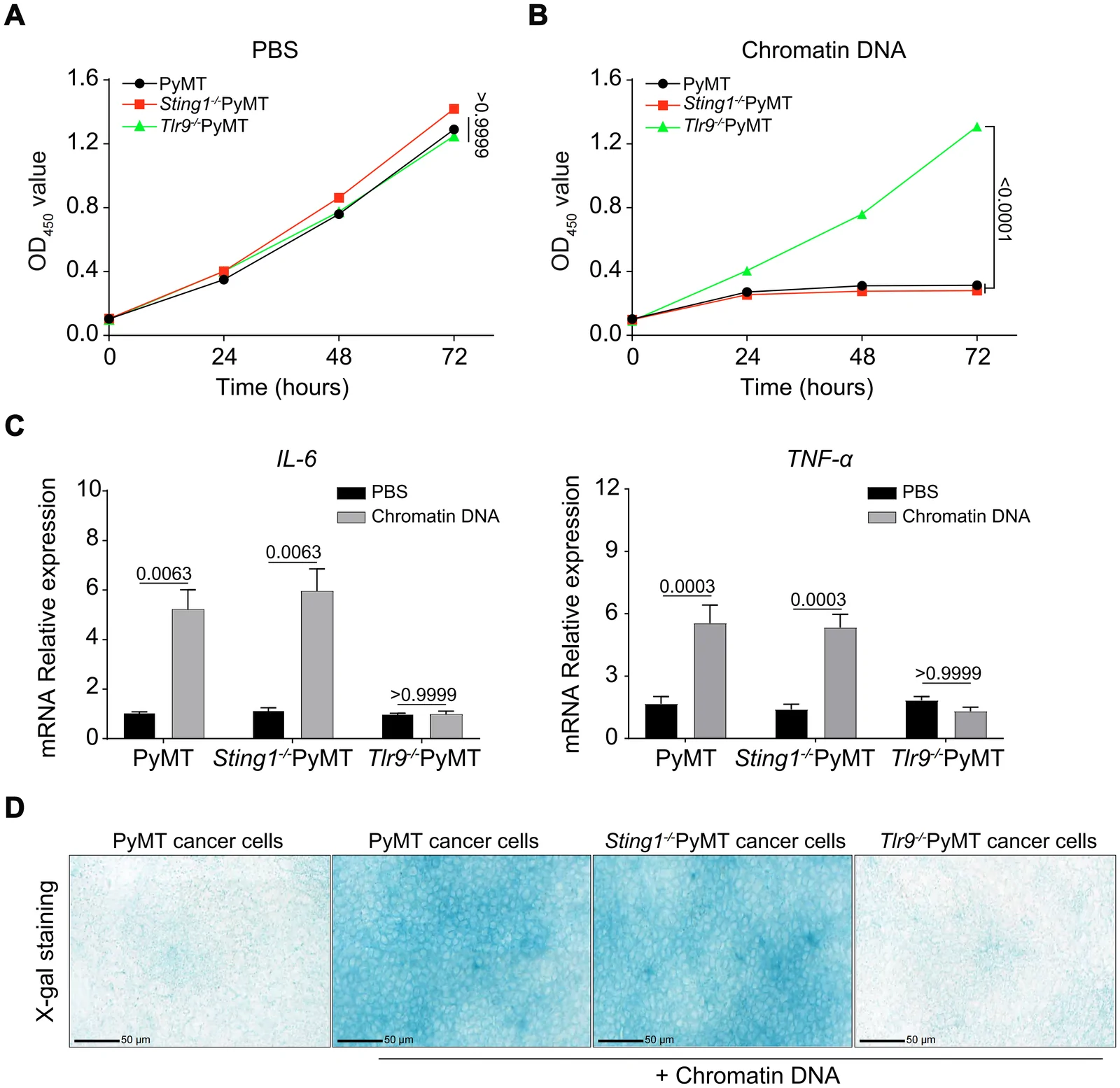
The *ex vivo* study of cancer cells from wild-type, STING or TLR9 knockout PyMT mice. **(A, B)** CD45^-^EpCAM^+^ cancer cells sorted from PyMT, *Sting1^-/-^*PyMT and *Tlr9^-/-^*PyMT mice, were co-cultured with PBS **(A)** or chromatin DNA **(B)** for the indicated time points; Cancer cell proliferation were monitored by CCK8 assay. **(C)** Quantitative RT-PCR analysis of SASP (IL-6, TNF-α) mRNA expression of CD45^-^EpCAM^+^ cancer cells sorted from PyMT, *Sting1^-/-^*PyMT and *Tlr9^-/-^*PyMT mice, which were incubated with control (PBS) or chromatin DNA for 24 hours. **(D)** Representative images of β-gal staining in cancer cells sorted from PyMT, *Sting1^-/-^*PyMT and *Tlr9^-/-^*PyMT mice, which were cultured with control (PBS) or chromatin DNA for 1 week. Scale bars, 50 μm. Data are mean ± SEM **(A–C)**; One-way ANOVA with *post hoc* Bonferroni *t*-test **(A, B)** and two-tailed unpaired *t*-test **(C)**. Data are representative of three independent experiments **(A–D)**.

### 3.5 Transcriptional profile of senescence

Based on the above observations, we investigated the transcriptional program of extracellular DNA-triggered cancer cell senescence. We isolated CD45^-^EpCAM^+^ cancer cells from PyMT (P), *Dnase1l3*^-/-^PyMT (*DD*P), *Ighm*^-/-^PyMT (*MM*P) and *Dnase1l3*^-/-^*Ighm*^-/-^PyMT (*DDMM*P) mice, and performed RNA-seq experiments. Compared with wild-type and single knockout counterparts, cancer cells from *Dnase1l3*^-/-^*Ighm*^-/-^PyMT mice upregulated 329 genes and downregulated 296 genes (**Supplementary Figure 5A**; **Supplementary Table 1**). Among these, a set of transcripts encoding NF-κβ signaling molecules (*Nfkb1*, *Nfkb2*, *Nfkbia*, *Nfkbid*, *Relb*) and p38/MAPK signaling factors (*Mapk6*, *Mapk8*, *Mapk3k2*, *Mapk3k8*) were upregulated in the cancer cells from *Dnase1l3*^-/-^*Ighm*^-/-^PyMT mice (**Figure 8A**), implying TLR9 downstream NF-κB and MAPK pathways activation (30, 31). In line with the RNA-seq results, the *in vitro* assays verified that NF-κβ and MAPK pathways were activated in response to chromatin DNA treatment and abolished in the context of TLR9 deficiency (**Supplementary Figure 5B**). Furthermore, the SASP profile induced by extracellular DNA comprises a complex mix of factors including not only the inflammatory cytokines (*Il1b*, *Il6*, *Tnfa*) previously shown in the RT-PCR results (**Figure 4D**), but also other SASP factors, such as growth factors (*Csf1*, *Csf3*), chemokines (*Ccl2*, *Ccl4*, *Cxcl1*, *Cxcl2*, *Cxcl5*), and matrix metalloproteinases (*Mmp8*, *Mmp9*), which were reported in previous reports (32–37). Gene set enrichment analysis (GSEA) further revealed that the upregulated transcripts were collectively overrepresented as NF-κβ and inflammatory gene signatures (**Figure 8A**). Consistent with the senescence phenotype, myogenesis, cellular metabolism and proliferation in cancer cells from *Dnase1l3*^-/-^*Ighm*^-/-^PyMT mice were inhibited compared to their counterparts, as revealed by the downregulated transcripts and reduced representation of gene signatures related to collagen and PI3K/AKT/mTOR signaling (**Figure 8B**). Collectively, MAPK and NF-κB pathways, as well as the SASP profile were the most highly enriched terms among the upregulated genes (**Figure 8C**). In addition, volcano plots indicated that *Dnase1l3* and *Ighm* double knockout versus wild-type identified large numbers and magnitudes of transcriptional changes that are statistically significant, while neither of the single knockout counterparts versus wild-type could do so, suggesting the synergistic effect of *Dnase1l3* and *Ighm* deficiency in inducing senescence (**Figure 8D**; **Supplementary Figures 5C, D**). Together, these observations suggest that extracellular DNA induces a TLR9-mediated transcriptional program leading to senescence in the context of *Dnase1l3* and *Ighm* double deficiency.

**Figure 8:**
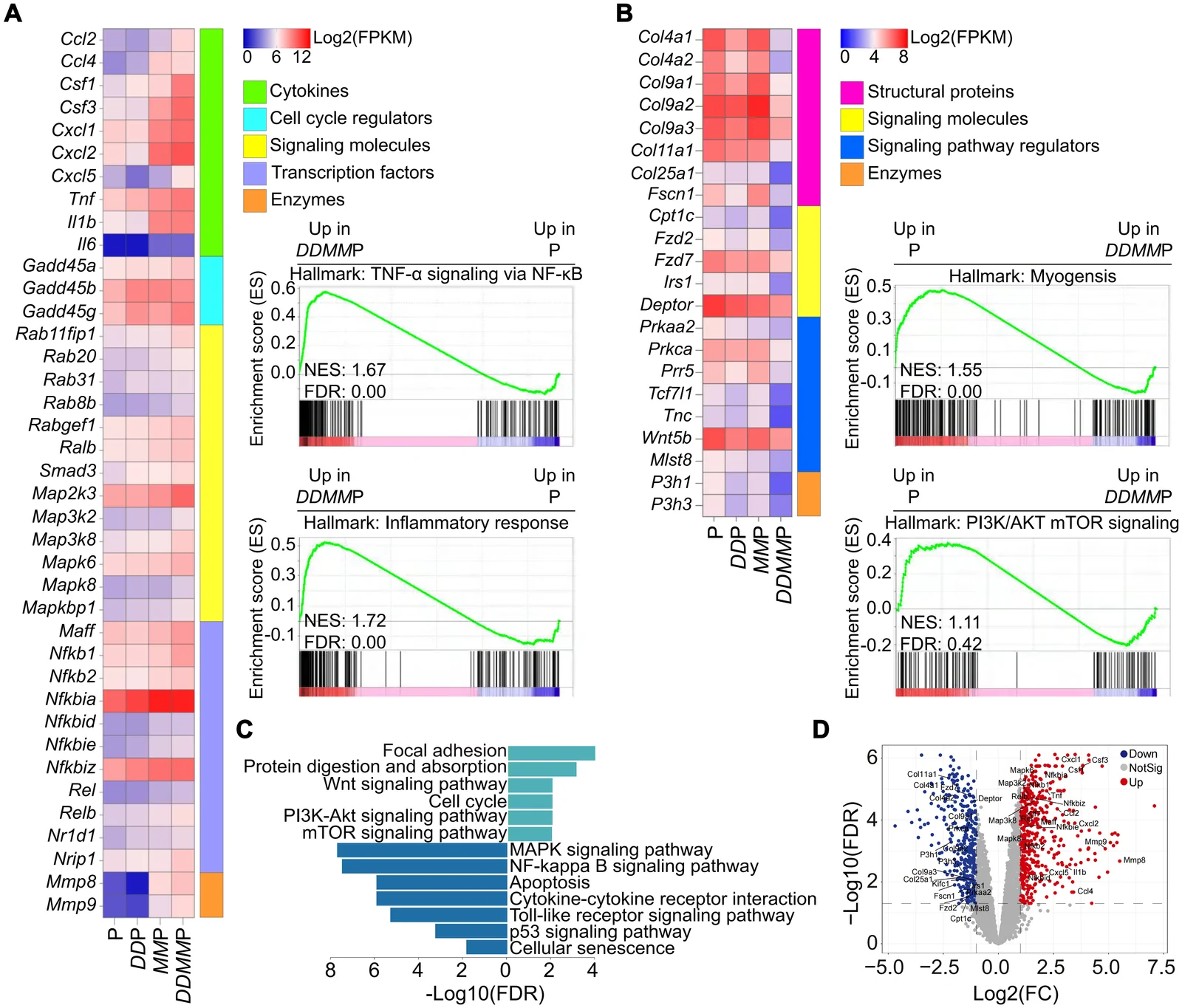
TLR9-induced gene signature in cancer cells. **(A, B)** Transcriptome of CD45^-^EpCAM^+^ cancer cells sorted from PyMT **(P)**, *Dnase1l3^-/-^*PyMT (*DD*P), *Ighm^-/-^*PyMT (*MM*P) and *Dnase1l3^-/-^Ighm^-/-^*PyMT (*DDMM*P) mice. The mean fragment per kilobase transcript per million mapped reads (FPKM) value of differentially expressed genes that were upregulated **(A)** or downregulated **(B)** in *DDMM*P, *DD*P or *MM*P group vs P group were calculated and plotted. The heatmap shows the ratio among mean FPKM value of each group and average of mean FPKM values of all four groups; The Gene Set Enrichment Analysis (GSEA) determines the defined set of genes showing the statistically significant, concordant differences between P group and *DDMM*P group. **(C, D)** KEGG pathway and volcano plot analysis between P group and *DDMM*P group.

## 4 Discussion

Extracellular chromatin DNA clearance by Dnase1l3 degradation and anti-DNA antibody-mediated phagocytosis operates in tumors to prevent foreign DNA from invading cancer cells. Even though cancer cells have engulfed the foreign chromatin DNA when Dnase1l3 and anti-DNA antibodies are unavailable, the endolysosomal TLR9 will sense the DNA and activate the downstream senescence-associated innate immunity, including NF-κB and p38/MAPK pathway activation, SASP factors secretion and CDKi proteins induction, thus ceasing cancer cell proliferation to maintain genome integrity (**Figure 9**). Our study reveals the TLR9 signaling linking extracellular DNA sensing to epithelial cell senescence, which extends the scope of TLR9-mediated innate immunity. TLR9 signaling is induced mainly by unmethylated CpG DNA sequences predominantly of bacterial origin (1), but such sequences can also be generated through demethylation of mammalian DNA in cancer cells. Demethylation of oncogenes and genome-wide DNA hypomethylation are associated with tumorigenesis (38, 39). In addition, increasing evidence indicates that TLR9 can be activated by mammalian self-DNA of nuclear origin (40, 41).

**Figure 9:**
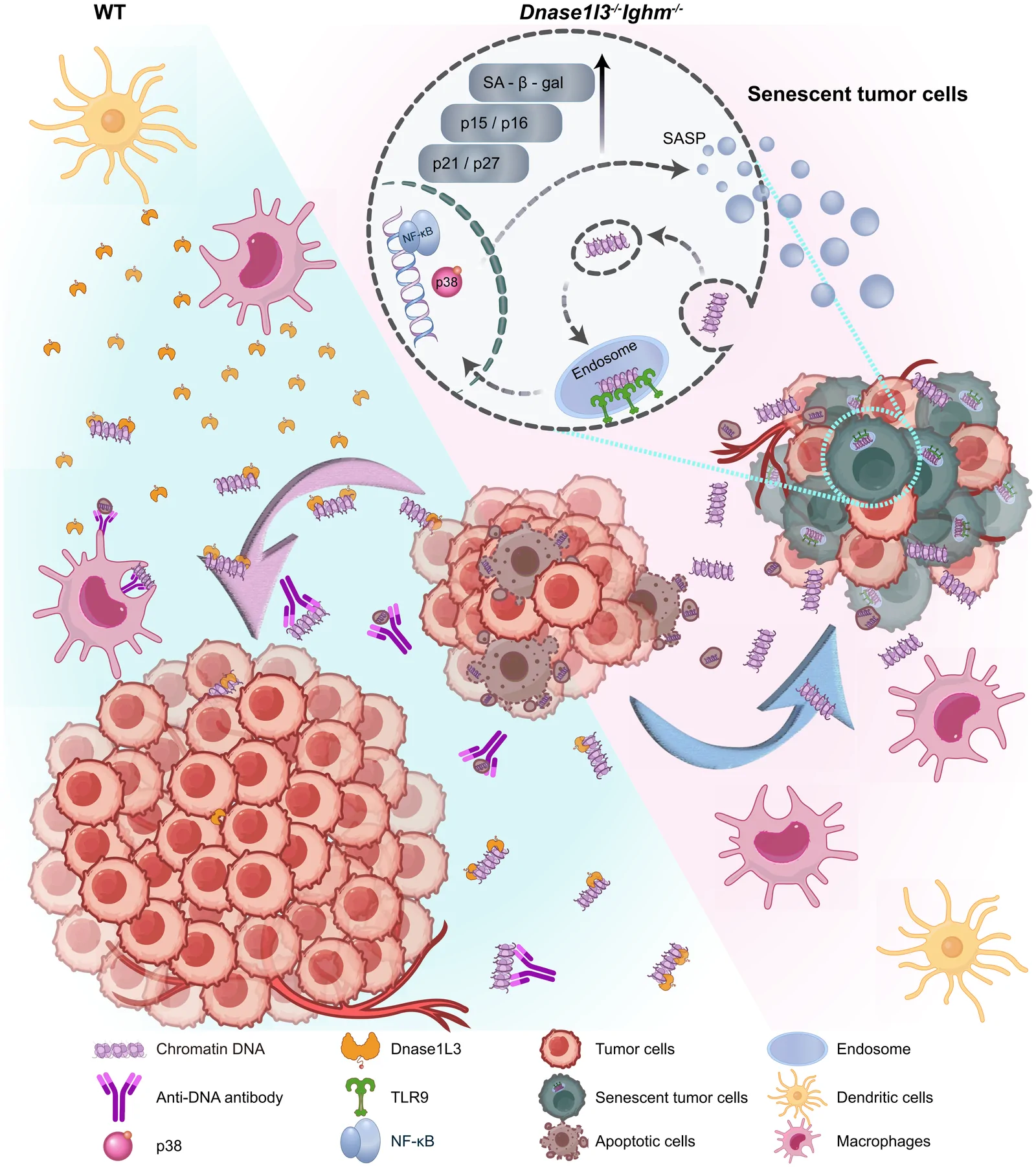
Clearance of extracellular chromatin DNA prevents senescence-associated innate immunity. Tumor progression is associated with excessive cancer cell proliferation and death, with chromatin DNA release from the apoptotic cancer cells being immunostimulatory. Hence, efficient clearance of extracellular chromatin DNA is crucial for cancer cells to evade immune defense. In the context of wild-type tumor, mononuclear phagocytes (Dendritic cells and macrophages)-derived Dnase1l3, collaborates with B cells-secreted autoreactive anti-DNA antibody to clear the extracellular chromatin DNA. In the case of *Dnase1l3* and *Ighm* double deficiency, cancer cells are forced to engulf extracellular chromatin DNA, which is sensed by endolysosomal TLR9, and triggers downstream NF-κB and p38 activation, SASP secretion, and thus leads to cancer cell senescence and profound repression of tumor progression.

Previous studies have attributed the pathogenic potential to anti-DNA antibodies in SLE patients (42). However, our study demonstrated that the production of anti-DNA autoantibody induced by Dnase1l3 deficiency, in essence, is a backup mechanism for extracellular DNA clearance. Furthermore, TLR9-mediated senescence serves as a backup for Dnase1l3 and anti-DNA autoantibody double deficiency. Generally, these multi-layered DNA scavenging pathways operate in tumors, with Dnase1l3- and anti-DNA autoantibody-assisted DNA clearance by hematopoietic lineage phagocytes superseding the TLR9-mediated blockade of DNA regeneration by epithelial lineage cancer cells; and the latter route effectively induces cancer cell dormancy. Notably, high tumor TLR9 expression is associated with significantly extended disease-specific survival in patients with triple negative breast cancer or renal clear cell carcinoma (43, 44).

Several limitations merit consideration. First, the employed Cre-driver lines exhibit imperfect lineage specificity. *MMTV-cre*, despite being designed for mammary epithelium targeted recombination, is also active in a subset of hematopoietic stem and progenitor cell compartments (45), which may result in unintended gene deletion within hematopoietic populations. CD11c is not exclusively restricted to dendritic cells, and Cx3cr1 is not confined solely to macrophages (46, 47). Consequently, neither *Cd11c-cre* nor *Cx3cr1-cre* can unequivocally separate macrophages from dendritic cells. We therefore cannot formally exclude a contribution of Cre-expressing non-target lineages to the phenotypes observed. Second, the group sizes for the Cx3cr1-cre and Cd11c-cre compound genotypes in **Figure 6F** were small (n = 3 per group), these findings should be validated in larger cohorts. Third, the RT-qPCR gene expression analysis relied on the 2^−ΔΔCt^ method. Although melting-curve analyses confirmed specific single amplicons, direct primer amplification-efficiency validation was not performed. Therefore, these qPCR data should be interpreted as relative expression trends instead of strict absolute quantitation.

In summary, our study unveils a multi-layered DNA scavenging pathway in tumors: primary clearance by Dnase1l3 and anti-DNA autoantibodies via phagocytes, and a backup TLR9-driven senescence response that halts DNA regeneration in cancer cells. This pathway extends TLR9 biology to epithelial senescence, reframes anti-DNA antibodies as protective rather than purely pathogenic, and clarifies autoimmunity as a natural protective response against foreign insults. Notably, although TLR9-mediated senescence is intrinsically an immune defense against genomic insults, our findings suggest that its therapeutic induction—by eliminating both Dnase1l3 and anti-DNA autoantibodies—could offer a cancer immunotherapeutic strategy by driving tumor cells into TLR9-dependent senescence and dormancy. Given that systemic Dnase1l3 inhibition risks autoimmunity and efficacy depends on tumor TLR9 expression, such an approach remains to be validated in future studies.

## Data Availability

Raw RNA-seq data from this study are deposited in NCBI SRA (accession PRJNA1538726, https://www.ncbi.nlm.nih.gov/sra/PRJNA1538726).
