# Supplementary material for: Clearance of extracellular DNA prevents senescence-associated antitumor immunity
Source: Front Immunol. 2026 Oct 14;17:1965273. doi: 10.3389/fimmu.2026.1965273 (PMC13714587; doi:10.3389/fimmu.2026.1965273)
Supplement: Supplementary file 1 [file SupplementaryFile1.docx]

**Supplementary Figure Legends**

# Supplementary Figure 1 Origin of Dnase1L3 and the effect of Dnase1L3 deficiency on tumor tissue. (A) Representative immunofluorescence images of E-cadherin (green), Dnase1L3 (red), F4/80 (white) and Vcam-1 (magenta) in tumor tissues from PyMT mice, and F4/80^+^Vcam-1^+^ cells indicate tumor-associated macrophages (TAMs). (B) Representative immunofluorescence images of E-cadherin (green), Dnase1L3 (red), F4/80 (white) and CD11c (yellow) in tumor tissues from PyMT mice, and CD11c^+^F4/80^-^ cells indicate dendritic cells (DCs). (C) Representative immunofluorescence images of E-cadherin (green), CD31 (red) and Cleaved caspase-3 (white) in tumor tissues from PyMT and *Dnase1l3^-/-^*PyMT mice. Scale bars, 25 μm and 5 μm. Data are representative of three independent experiments (A, B, C).

**Supplementary Figure 2 Generation and functional analysis of recombinant 3H9 anti-DNA IgG2a and its controls.** (A) Analysis of serum anti-DNA and anti-nucleosome IgG subclasses in *Dnase1l3^-/-^*PyMT mice (n=5). (B) Schematic of antibody structure for Con IgG2a, 3H9 IgG2a, and 3H9 IgG2a-LALAPG. (C) Assessment of Con IgG2a, 3H9 IgG2a and 3H9 IgG2a-LALAPG binding to DNA, Chromatin, mFcγRI (mouse FcγRI) and mFcγRIII (mouse FcγRIII) by ELISA. Value of optical density (OD) was detected at 450 nm. (D) ELISA to assess the serum levels of Con IgG2a, 3H9 IgG2a and 3H9 IgG2a-LALAPG post i.v. injection at the indicated time. Data are mean ± SEM (A, C, D). Data are pooled biological replicates (A) or representative of three independent experiments (C, D).

**Supplementary Figure 3 The effect of *Dnase1l3* and *Ighm* double knockout on cancer cell death and cell cycle proteins.** (A) Representative immunofluorescence images of DAPI (blue), E-cadherin (green) and Cleaved caspase-3 (white) in tumor tissues from PyMT, *Dnase1l3^-/-^*PyMT, *Ighm^-/-^*PyMT and *Dnase1l3^-/-^Ighm^-/-^*PyMT mice. (B) Immunoblot of CDK (CDK2, CDK4) and Cyclin (CyclinD1, CyclinE) protein expression in CD45^-^EpCAM^+^ cancer cells sorted from PyMT, *Dnase1l3^-/-^*PyMT, *Ighm^-/-^*PyMT and *Dnase1l3^-/-^Ighm^-/-^*PyMT mice. Scale bars, 25 μm. Data are representative of three independent experiments (A, B)

**Supplementary Figure 4 Association of senescence phenotype with tumor inhibition.** (A) Representative images of β-gal staining in tumor tissue from PyMT and *Dnase1l3^-/-^*PyMT mice treated with control IgG (Con IgG) or anti-CD20 IgG (αCD20 IgG). (B) Representative images of β-gal staining in tumor tissue from *Dnase1l3^-/-^Ighm^-/-^*PyMT mice treated with PBS, anti-DNA IgG2a (3H9 IgG2a), control IgG2a (Con IgG2a) or anti-DNA IgG2a with LALAPG mutation (3H9 IgG2a-LALAPG). (C) Representative images of β-gal staining in tumor tissue from *Dnase1l3^-/-^*PyMT mice treated with PBS, Con liposome or Clophosome. The percentage of β-gal^+^ cells over total cells was calculated from 0.04-mm^2^ regions (n = 8 for each group). Scale bars, 25 μm. Data are mean ± SEM (A, B, C). Two-way ANOVA with post hoc Bonferroni *t*-test (A, B, C). Data are representative of three independent experiments (A, B, C).

**Supplementary Figure 5 Differentially expression gene analysis between cancer cells from different cohorts.** (A) Comparing the overlap between sets of differentially expressed genes (DEGs) in *DDMM*P vs P, *DD*P vs P and *MM*P vs P; RNA-seq of CD45^-^EpCAM^+^ cancer cells sorted from PyMT (P), *Dnase1l3^-/-^*PyMT (*DD*P), *Ighm^-/-^*PyMT (*MM*P) and *Dnase1l3^-/-^Ighm^-/-^*PyMT (*DDMM*P) mice was done and differentially expressed genes analysis was performed. (B) Immunoblot of the indicated protein expression in the CD45^-^EpCAM^+^ cancer cells (sorted from PyMT and *Tlr9^-/-^*PyMT mice) treated with PBS (Mock) or chromatin DNA. (C) Volcano plot of *DD*P (*Dnase1l3^-/-^*PyMT) group vs P (PyMT) group. (D) Volcano plot of *MM*P (*Ighm^-/-^*PyMT) group vs P (PyMT) group. Data are representative of three independent experiments (B).
